# Treatment of type 2 diabetes mellitus using the traditional Chinese medicine Jinlida as an add-on medication: A systematic review and meta-analysis of randomized controlled trials

**DOI:** 10.3389/fendo.2022.1018450

**Published:** 2022-10-17

**Authors:** Xuemin Zhao, Linfei Liu, Jing Liu

**Affiliations:** ^1^ Department of Internal Medicine, Chengde Medical University, Chengde, China; ^2^ Sericultural Research Institute, Chengde Medical University, Chengde, China; ^3^ Department of Cardiovascular Medicine, The Second Affiliated Hospital of Shaanxi University of Chinese Medicine, Xianyang, China

**Keywords:** Jinlida granule, add-on therapy, type 2 diabetes, syndrome differentiation, qi-yin deficiency

## Abstract

**Background:**

Numerous randomized controlled trials (RCTs) conducted in China have shown that jinlida granules are a promising traditional Chinese medicine (TCM) for the treatment of persons with type 2 diabetes mellitus (T2DM). Controversial results have been reported in different RCTs. The aim of our study was to evaluate the adjuvant hypoglycemic effect of jinlida granules on persons with T2DM and to explore the source of heterogeneity between these RCTs.

**Materials and methods:**

Medical article databases were individually searched by two authors for RCTs that provided data regarding the effect of jinlida granules in the treatment of T2DM before 1 June 2022. The methodological quality of the included RCTs was comprehensively assessed by two authors. Data from RCTs with low risk of bias were pooled using Stata SE 12.0 (random-effects model). Evidence derived from the meta-analysis will be assessed according to the GRADE system.

**Results:**

Twenty-two RCTs were eventually included in the systematic review and three RCTs with low risk of bias were analyzed in the meta-analysis. Compared with the control groups, significant changes were found in lowering glycosylated hemoglobin a1c (mean difference -0.283 with 95% CI -0.561, -0.004; *P*=0.046), and were not found in lowering 2-hour postprandial glucose (mean difference -0.314 with 95% CI -1.599, 0.972; *P*=0.632) and fasting blood glucose (mean difference -0.152 with 95% CI -0.778, -0.474; *P*=0.634) in the jinlida groups. The GRADE-assessed evidence quality for the outcomes was moderate.

**Conclusion:**

The adjuvant hypoglycemic effect of jinlida granules on adult Chinese persons with T2DM was statistically found in lowering HbA1c and was not statistically found in lowering FPG and 2h-PG. Evidence grading should be considered moderate, and the results should be interpreted cautiously. Whether the efficacy of HbA1c-lowering related to clinical significance remains to be investigated in future RCTs. Differences in HbA1c, FPG and 2h-PG at baseline and high risk of bias were important source of heterogeneity between these RCTs. In order to objectively evaluate the efficacy of jinlida granules on T2DM, it is urgently needed that high-quality RCTs evaluating the hypoglycemic effect of jinlida granules in the treatment of qi-yin deficiency pattern T2DM.

**Systematic review registration:**

https://www.crd.york.ac.uk/prospero/, identifier CRD42018085135.

## 1 Introduction

Diabetes mellitus, 90-95% of which is accounted for type 2 diabetes mellitus (T2DM), is an important cause of mortality and morbidity. The prevalence of diabetes mellitus continues to increase worldwide and in China. It is estimated that there were approximately 462 million persons with T2DM worldwide in 2017 (1, 2). The estimated prevalence of diabetes mellitus in adults living in mainland China was 11.2% to 12.8% in 2017 (3). Despite the efforts of the Western medicine, the increasing incidence of T2DM has indicated that available treatments are insufficient to reduce the prevalence of diabetes mellitus (4).

TCM has been universally used in China for thousands of years to treat diabetes mellitus through the dialectical approach of TCM (5). The curative effect of TCM in the prevention and treatment of T2DM has been comprehensively recognized in China (6). Studies of Chinese herbal medicine, especially those published in English journals, have opened up a new path for the treatment of T2DM and the efficacy of Chinese herbal medicine should be investigated and verified further (7, 8). According to syndrome differentiation in TCM, there are several subtypes of T2DM, such as yin-yang deficiency pattern T2DM, qi-yin deficiency pattern T2DM (QYDT2DM), yin deficiency and dry heat pattern T2DM, and other patterns (6, 9). Jinlida granules (JinLiDaKeLi), which are considered suitable for persons with QYDT2DM, are a traditional Chinese patent medicine (Shijiazhuang Yiling Pharmaceutical Co.) made of 17 herbs (Ginseng, rhizoma polygonati, dogwood, rehmanniae, anemarrhena, ophiopogon japonicus, polygoni multiflori, cortex lycii radices, coptis chinensis, salvia, rhizoma atractylodis lanceae, poria perrin, puerariae, semen litchi, epimedium and sophorae flavescentis) (10).

An animal study indicated that jinlida granules could alleviate metabolic disorders and ameliorate dysfunction of the hypothalamic-pituitary-thyroid axis (11, 12). Numerous RCTs in China have shown that jinlida granules significantly reduce FPG, 2h-PG and HbA1c in persons with T2MD (13–17). A system review and meta-analysis of RCTs also showed that jinlida granules statistically and clinically lower FPG, 2-h PG, and HbA1c in persons with T2DM (18). However, significant changes in the main outcomes (HbA1c, FPG, 2-h PG) were not shown between the jinlida combined with metformin group and the metformin group in a newly published RCT (19). The aim of our study was to evaluate the adjuvant hypoglycemic effect of jinlida granules on persons with T2DM and to explore the source of heterogeneity between these RCTs.

## 2 Materials and methods

### 2.1 Systematic review registration

The protocol of our study was registered on 17 January 2018, and the registration number in PROSPERO is CRD42018085135. (https://www.crd.york.ac.uk/prospero/).

### 2.2 Search Strategy

Chinese databases (CNKI and Wan-fang) and English databases (PubMed, the Cochrane Library, Embase) were independently searched by two investigators (Zhao X and Liu L). Investigators searched the databases for articles published before 1 June 2022 using the following terminology: diabetes, jinlida, randomized, random and the corresponding Chinese terminology for Chinese databases. The search strategy for databases was [random(Text Word)] OR [randomized(Text Word)] AND [diabetes(Abstract)] AND [jinlida(Abstract)]. Other reports were identified by examining the references of the obtained articles. The PRISMA reporting guidelines were followed in our study.

### 2.3 Selection criteria

**Figure 1:**
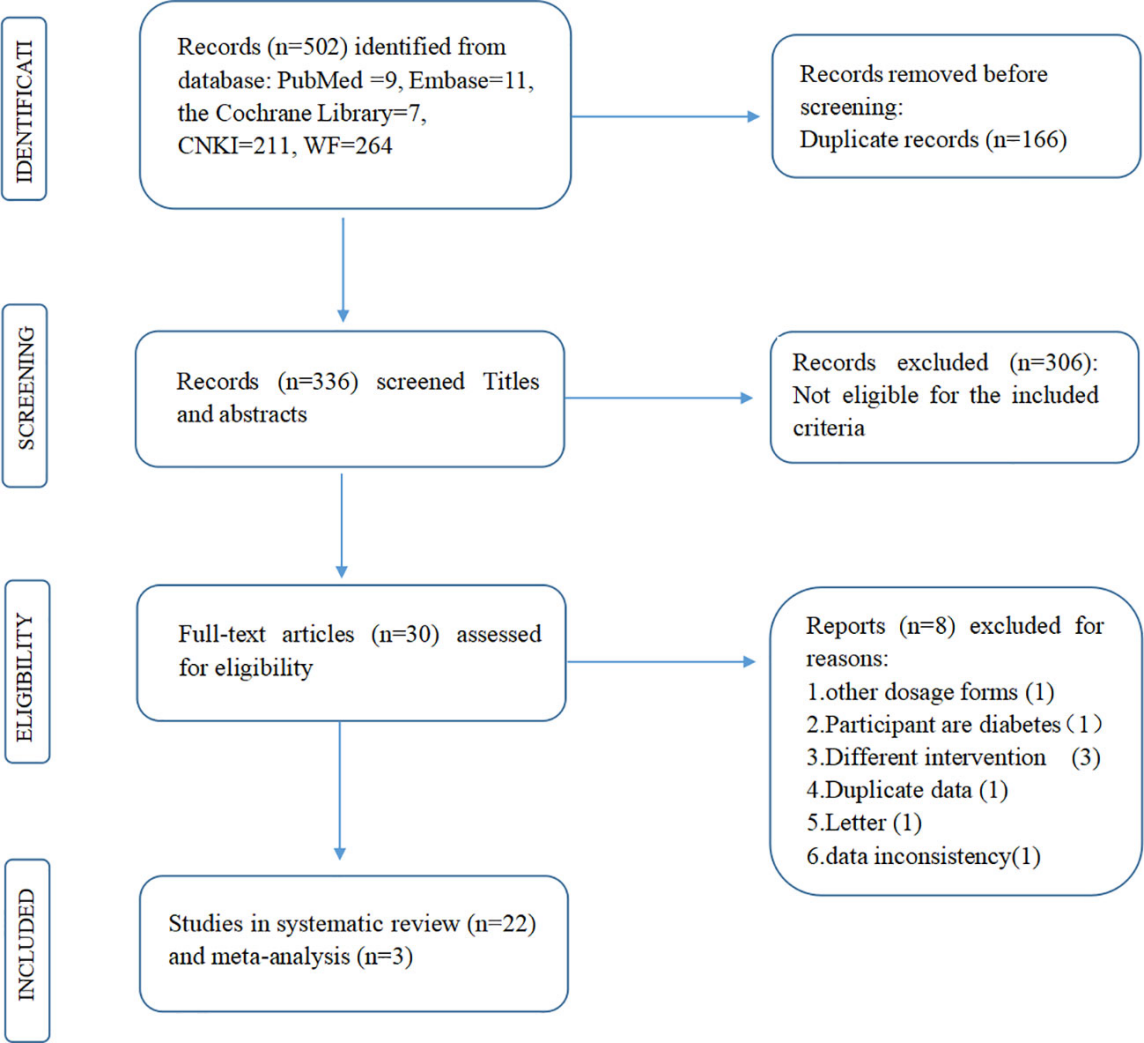
Flowchart of the study’s selection process.

The titles and abstracts of all acquired reports were first screened (Zhao X and Liu L). Studies fulfilling the following criteria (PICOS) were included: (1) the study recruited adult T2DM participants without severe renal or hepatic insufficiency or severe complications of diabetes; (2) the intervention was jinlida granules with metformin, and the comparison was metformin; (3) the primary outcomes were FPG, 2-h PG, and HbA1c after treatment; and (4) the study design was an RCT to lower the risk of bias. RCTs fulfilling the following criteria were excluded: (1) animal studies, case reports, meta-analyses, reviews, conference proceedings, and letters. (2) The number of participants in both groups and the levels of FPG, 2-h PG, and HbA1c (mean ± standard deviation) after treatment were not available. (3) There were significant differences in baseline characteristics between the jinlida group and the control group. (4) The participants in the RCTs were pregnant and lactating. Then, full-text reading was completed according to the given selection criteria (**Figure 1**).

### 2.4 Data extraction

Two authors (Zhao X and Liu L) independently extracted necessary information and data on outcomes from eligible RCTs (**Table 1**); the extracted data were as follows: author name, number of participants in each group (n), syndrome differentiation of TCM, age of participants, interventions, comparison, outcomes, and duration. If necessary data were not available in the RCTs, we contacted the corresponding author to request the missing data.

**Table 1:** The main information and risk of bias assessment of the included RCTs.

Author	Year	Age^*^I(n);C(n)	SDM	Interventions (usage)	Comparison (usage)	Duration	Risk of Bias Assessment (ROB)							
							RSG	AC	PB	DB	AB	RB	OB	Overall
Pan	2021	54.57 ± 10.54(33); 56 ± 9.47(36)	N.A.	Metformin (0.5g tid)+ Jinlida granule (9g tid)	Metformin (0.5g tid)+ placebo (9g tid)	16 weeks	Low	Low	Low	Low	Low	Low	Unclear	Low
Lian	2015	55.18 ± 9.13(92); 55.81 ± 9.93 (94)	N.A.	Metformin(0.5g bid)+ Jinlida granule (9g tid)	Metformin (0.5g bid) +placebo (9g tid)	12 weeks	Low	Low	Low	Low	Low	Low	Unclear	Low
Zhao	2017	51.76 ± 8.88(78); 49.44 ± 10.93(78)	N.A.	Metformin(0.5g tid)+ Jinlida granule (9g tid)	Metformin (0.5g tid)+ placebo (9g tid)	12 weeks	Low	Low	Low	Low	Low	Unclear	Unclear	Low
Wang	2016	54.28 ± 9.23(90); 54.71 ± 9.83(90)	N.A.	Metformin(1.5g/day)+ Jinlida granule (9g tid)	Metformin(2g/day)	12 weeks	Low	Unclear	High	High	Low	Unclear	Unclear	High
Xing	2021	57.58 ± 2.13(50); 57.7 ± 2.24(50)	N.A.	Metformin(0.25g tid)+ Jinlida granule(9g tid)	Metformin(0.25g tid)	3month	Low	Unclear	High	High	Low	Unclear	Unclear	High
Zhang	2019	47.25 ± 5.17(61); 46.61 ± 5.08(61)	N.A.	Metformin(0.5g tid)+ Jinlida granule (9g tid)	Metformin(0.5g tid)	12 weeks	Low	Unclear	High	High	Low	Unclear	Unclear	High
Wu	2019	45.14 ± 6.13(56); 46.32 ± 5.55(56)	QYD	Metformin(0.5g tid)+ Jinlida granule (9g tid)	Metformin(0.5g tid)	3month	Low	Unclear	High	High	Low	Unclear	Unclear	High
Liu	2017	69.1 ± 8.2 (60); 68.6 ± 7.1 (60)	N.A.	Metformin(0.5g tid)+ Jinlida granule (9g tid)	Metformin(0.5g tid)	8 weeks^&^	Low	Unclear	High	High	Low	Unclear	Unclear	High
Li	2017	72.96 ± 2.87(46); 72.16 ± 2.14(46)	QYD	Metformin(0.5g tid)+ Jinlida granule(9g tid)	Metformin(0.5g tid)	3month#	Low	Unclear	High	High	Low	Unclear	Unclear	High
Zhou	2020	32.48 ± 2.28(39); 32.51 ± 2.25(39)	N.A.	Metformin(0.5g tid)+ Jinlida granule(9g tid)	Metformin(0.5g tid)	3month^&^	Unclear	Unclear	High	High	Low	Unclear	Unclear	High
Wang	2020	50.57 ± 8.1(65); 47.89 ± 6.72(65)	N.A.	Metformin(0.5g tid)+ Jinlida granule(9g tid)	Metformin(0.5g tid)	12 weeks	Unclear	Unclear	High	High	Unclear	Unclear	Unclear	High
Liu	2020	64.42 ± 5.31(40); 63.96 ± 5.42(40)	N.A.	Metformin(0.5g tid)+ Jinlida granule(9g tid)	Metformin(0.5g tid)	3month	Unclear	Unclear	High	High	Low	Unclear	Unclear	High
Zhang	2019	62.4 ± 5.9(30); 62.1 ± 6.1(30)	N.A.	Metformin(≥1g/day)+ Jinlida granule(9g tid)	Metformin(≥1g/day)	8 weeks^&^	Unclear	Unclear	High	High	Low	Unclear	Unclear	High
Liu	2018	48.26 ± 4.93(35); 47.14 ± 4.87(35)	N.A.	Metformin(0.5g bid)+ Jinlida granule (9g tid)	Metformin(0.5g tid)	8 weeks	Unclear	Unclear	High	High	Unclear	Unclear	Unclear	High
Wang	2017	56 ± 13.5(55); 57 ± 12.1(55)	N.A.	Metformin (0.5g tid)+ Jinlida granule (9g tid)	Metformin (0.5g tid)	60days	Unclear	Unclear	High	High	Low	Unclear	Unclear	High
Zheng	2016	46 ± 4(74); 48 ± 5(74)	N.A.	Metformin(0.5-1g/day) +Jinlida granule((9g tid)	Metformin(0.5-1g/day)	3month	Unclear	Unclear	High	High	Low	Unclear	Unclear	High
Yue	2016	40-63(30); 43-74(30)	N.A.	Metformin (N.A.)+ Jinlida granule (9g tid)	Metformin (N.A.)	16 weeks	Unclear	Unclear	High	High	Unclear	Unclear	Unclear	High
Guo	2013	53.2 ± 9.4(44); 52.2 ± 9.5(44)	N.A.	Metformin (0.5g tid)+ Jinlida granule (9g tid)	Metformin (0.5g tid)	16 weeks	Unclear	Unclear	High	High	Low	Unclear	Unclear	High
Song	2017	55.1 ± 12.4(30); 56 ± 11.4(30)	N.A.	Metformin(0.5g tid)+ Jinlida granule(9g tid)	Metformin(0.5g tid)	3month	Low	Unclear	High	High	Low	Unclear	Unclear	High
Yuan	2018	45 ± 6(66); 46 ± 4(66)	N.A.	Metformin (N.A.)+ Jinlida granule(9g tid)	Metformin (N.A.)	4month	Unclear	Unclear	High	High	Low	Unclear	Unclear	High
Tang	2017	51.83 ± 12.61(35); 51.11 ± 11.44(35)	N.A.	Metformin(0.25g tid)+ Jinlida granule(9g tid)	Metformin(0.25g tid)	8 weeks	Unclear	Unclear	High	High	Low	Unclear	Unclear	High
Su	2014	30-70 (30); 30-70 (30)	QYD+ blood- stasis	Metformin (N.A.)+ Jinlida granule (9g tid)	Metformin (N.A.)	12 weeks	Unclear	Unclear	High	High	Low	Unclear	Unclear	High

SDM, syndrome differentiation of traditional Chinese medicine; n, sample size; I, interventions; C:comparison; N.A., not available; RSG, random sequence generation; AC, allocation concealment; PB, performance bias; DB, detection bias; AB, attrition bias; RB, reporting bias; OB, other bias; *Mean ± standard deviation (year); bid: two times daily; tid: three times daily; ^#^without outcome of PBG; ^&^without outcome of HbA1c. QYD, qi-yin deficiency pattern.

### 2.5 Quality assessment

The methodological quality assessment of the included RCTs was independently completed by two researchers (Zhao X and Liu L) in line with the Cochrane Risk of Bias Assessment Tool (20). Disagreements about the above process were resolved by consulting with the third author (Liu J).

### 2.6 Statistical analyses

The effect values of RCTs were analyzed in the meta-analysis using a random-effects model. Continuous data such as FPG, 2-h PG, and HbA1c (mean ± standard deviation) are described as weighted mean differences (WMDs) with 95% confidence intervals (CIs). The statistical heterogeneity among the RCTs was considered to be notable according to an *I* ^2^ value > 50%, and the source of heterogeneity was analyzed. Sensitivity analysis should be completed by eliminating studies individually. Publication bias tests (Begg’s and Egger’s) should also be performed (21, 22). Data processing in our meta-analysis was performed using statistical software (Stata SE 12.0).

### 2.7 The evidence grading (GRADE system)

The evidence grade of the outcome of our meta-analysis was determined using the Grading of Recommendations Assessment, Development and Evaluation (GRADE) guidelines (23).

## 3 Results

A total of 502 potential records were initially yielded. Of these records, 472 were excluded after reviewing the titles and abstracts due to lack of eligibility (n = 306) or duplicated reports (n = 166). Eight articles were excluded for various reasons (24–31). Finally, 22 RCTs were systematically reviewed (13–17, 19, 32–47).

### 3.1 Assessment of methodological quality of included RCTs

All included RCTs were performed in China with male and female adult participants. Nine RCTs described the random sequence method and only 3 RCTs described the method for concealing the allocation of the intervention group or the comparison group. only 3 RCTs were designed as double-blind, placebo-controlled trials. Ten RCTs described the loss of participants in the trials and only 2 RCTs have been registered in the China Clinical Trials Registry (http://www.chictr.org.cn). Nineteen RCTs were considered as high risk of bias and, only 3 RCTs were considered as low risk of bias according to the assessment tool of risk of bias. The main information and risk of bias assessment of the included RCTs are summarized in **Table 1**. The nineteen RCTs with high risk of bias were only described qualitatively instead of meta-analysis. Fourteen out of the 16 RCTs with high risk of bias indicated that jinlida has an adjuvant effect in reducing HbA1c (**Figure S1**). Twelve out of the 18 RCTs with high risk of bias indicated jinlida has the adjuvant effect in reducing 2h-PG (**Figure S2**). Sixteen out of the 19 RCTs with high risk of bias indicated jinlida has the adjuvant effect in reducing FPG (**Figure S3**). There seems to be a trend that the adjunctive effects of lowering HbA1c and 2h-PG become more pronounced with the year of publication. Only three RCTs with low risk of bias were pooled for outcomes in the subsequent meta-analysis.

### 3.2 Data analysis of outcomes and evidence grading

#### 3.2.1 Comparison of changes in HbA1c between Jinlida groups and control groups

**Figure 2:**
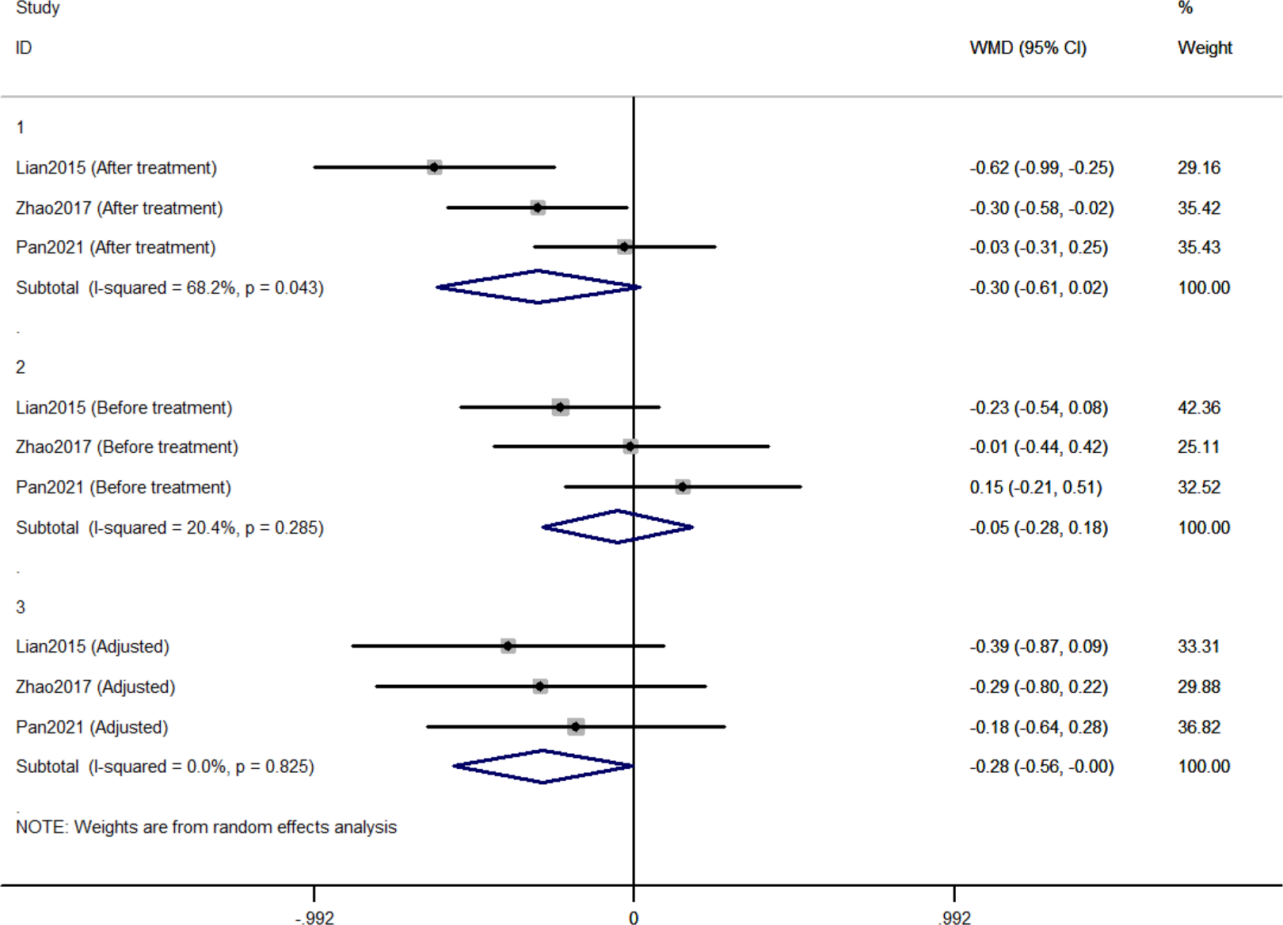
Forest plots of changes in HbA1c between Jinlida Groups and Control Groups.

Three RCTs included 411 individuals reported the effect of jinlida granules on HbA1c. The pooled weighted mean difference (WMD) of HbA1c after treatment was -0.298 (95% CI: -0.612, 0.017; *P*=0.064) with notable heterogeneity (*I* ^2 =^ 68.2%) (**Figure 2**). The pooled WMD of HbA1c before treatment was -0.051 (95% CI: -0.284, 0.181; *P*=0.666) without notable heterogeneity (*I* ^2 =^ 20.4%). The difference in HbA1c in the jinlida groups and control groups before treatment may be a source of heterogeneity. Then, the WMD of HbA1c after treatment in each RCT was adjusted according to the levels of HbA1c before treatment. The pooled adjusted WMD of HbA1c was -0.283 (95% CI: -0.561, -0.004; *P*=0.046) without notable heterogeneity (*I* ^2 =^ 0%). The adjusted WMD of HbA1c was consistent among the three RCTs. However, the results are imprecise and likely to be changed by future RCTs. The evidence grading of changes in HbA1c should be considered as moderate (**Table 2**).

**Table 2:** The evidence quality for the outcomes (GRADE system).

Outcomes (N)	AWMD (95% CI)	ROB	Inconsistency	Indirectness	Imprecision	Other considerations	Sample size		Evidence quality
							Interventions	Comparison	
HbA1c (3)	-0.283 (-0.561, -0.004)	none	none	none	serious	none	203	208	MODERATE **⊕⊕⊕**○
FBG (3)	-0.314 (-1.599, 0.972)	none	serious	none	serious	none	203	208	MODERATE **⊕⊕⊕**○
2h-PG (3)	-0.152 (-0.778, -0.474)	none	serious	none	serious	none	203	208	MODERATE **⊕⊕⊕**○

AWMD, adjusted weighted mean difference; ROB, risk of bias; N, numbers of RCTs; GRADE, Grading of Recommendations Assessment, Development and Evaluation

#### 3.2.2 Comparison of changes in FPG between Jinlida groups and control groups

**Figure 3:**
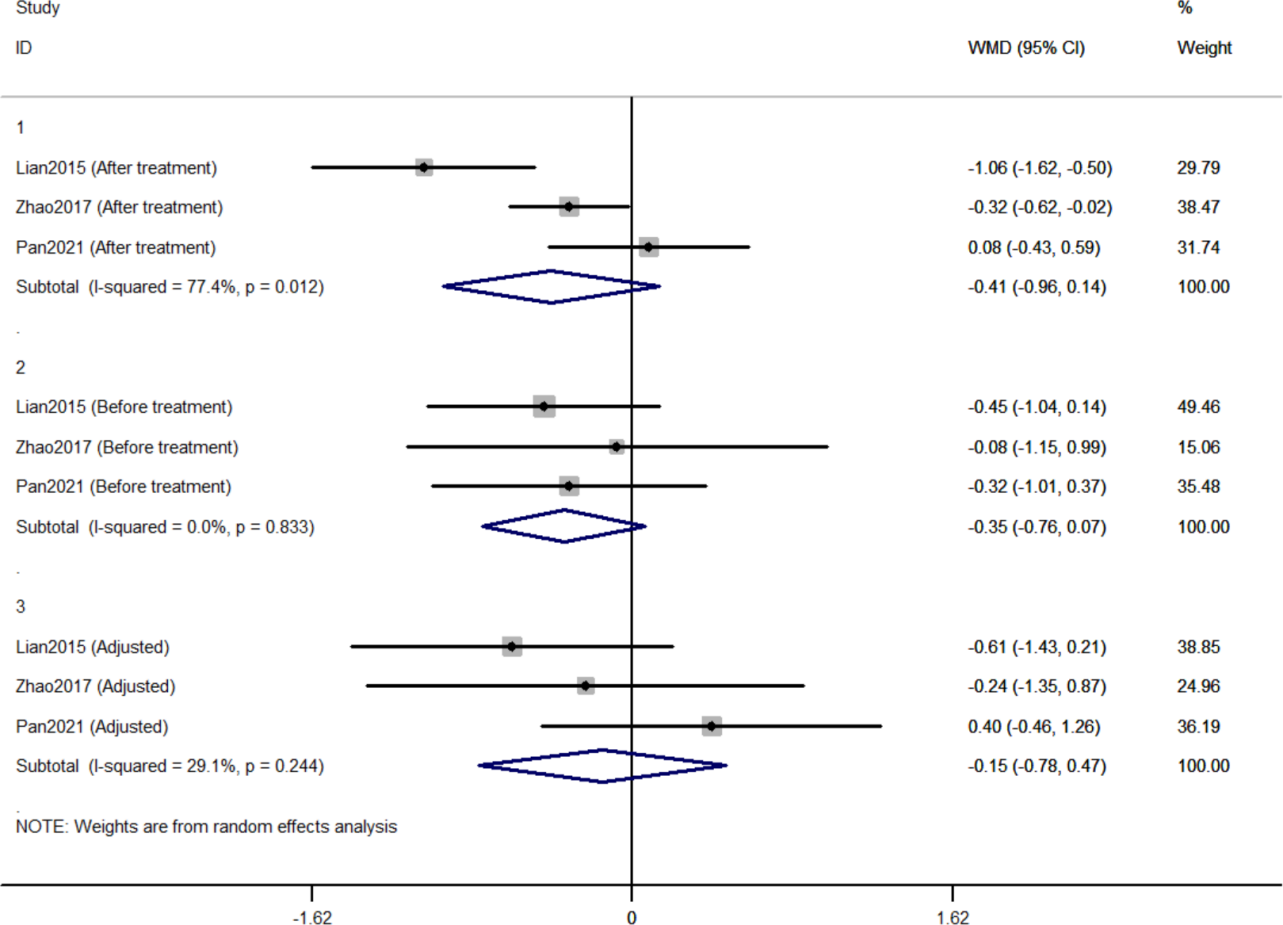
Forest plots of changes in FPG between Jinlida Groups and Control Groups.

Three RCTs included 411 individuals reported the effect of jinlida granules on FPG. The pooled WMD of FPG after treatment was -0.414 (95% CI: -0.962, 0.135) with notable heterogeneity (*I* ^2 =^ 77.4%) (**Figure 3**). The pooled WMD of FPG before treatment was -0.348 (95% CI: -0.762, 0.065; *P*=0.099) without notable heterogeneity (*I* ^2 =^ 0). The difference in FPG in the jinlida groups and control groups before treatment may be a source of heterogeneity. Then, the WMD of FPG after treatment in each RCT was adjusted according to the levels of FPG before treatment. The pooled adjusted WMD of FPG was -0.152 (95% CI: -0.778, 0.474; *P*=0.634) without notable heterogeneity (*I* ^2 =^ 29.1%). The adjusted WMD of FPG in the three RCTs was inconsistent, and the 95% CI of the pooled adjusted WMD is great range. The evidence grading of changes in FPG should also be considered as moderate (**Table 2**).

#### 3.2.3 Comparison of changes in 2h-PG between Jinlida groups and control groups

**Figure 4:**
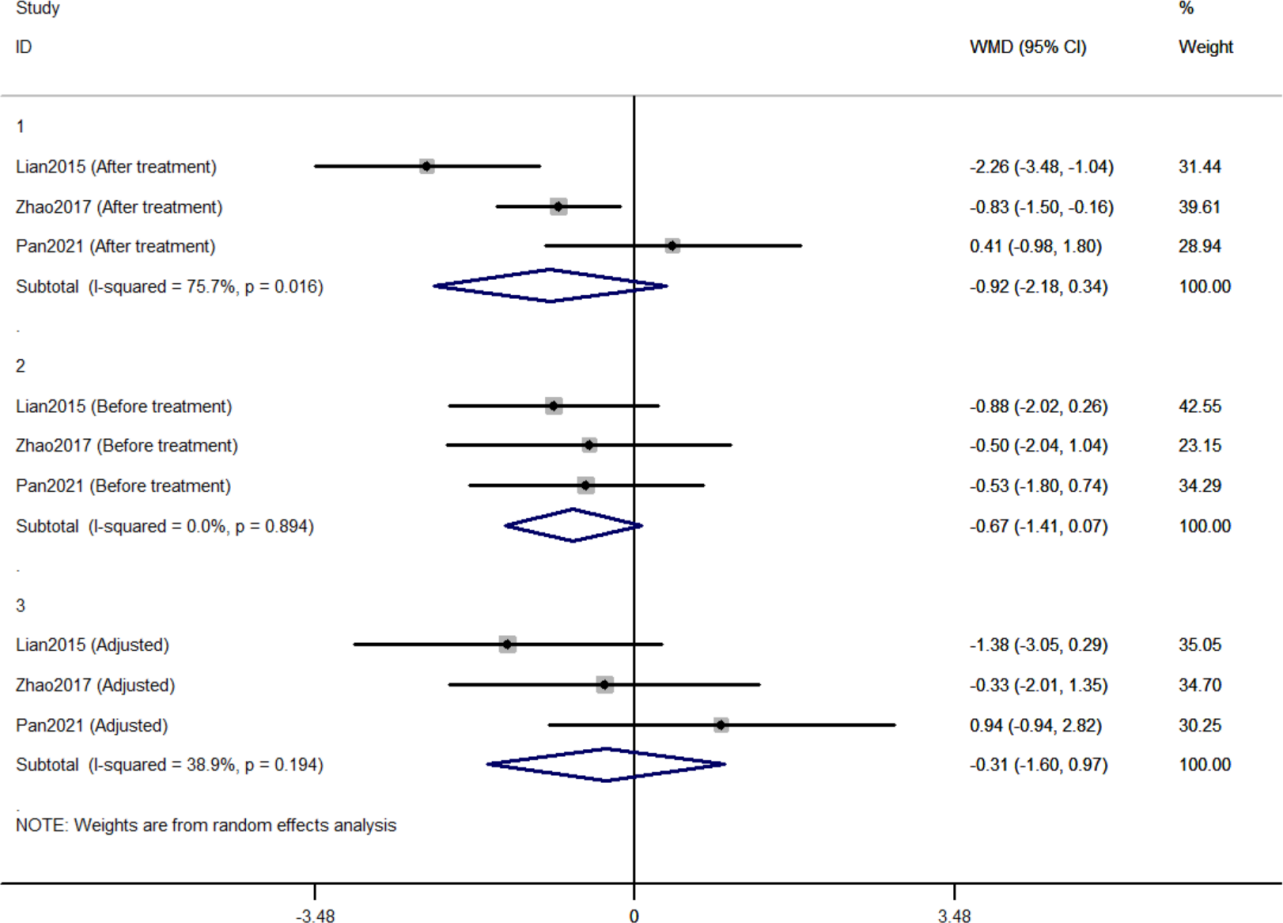
Forest plots of changes in 2h-PG between Jinlida Groups and Control Groups.

Three RCTs included 411 individuals reported the effect of jinlida granules on 2h-PG. The pooled WMD of 2h-PG after treatment was -0.921 (95% CI: -2.179, 0.338; *P*=0.152) with notable heterogeneity (*I* ^2 =^ 75.7%) (**Figure 4**). The pooled WMD of 2h-PG before treatment was -0.672 (95% CI: -1.415, 0.071; *P*=0.076) without notable heterogeneity (*I* ^2 =^ 0). The difference in 2h-PG in the jinlida groups and control groups before treatment may be a source of heterogeneity. Then, the WMD of 2h-PG after treatment in each RCT was adjusted according to the levels of 2h-PG before treatment. The pooled adjusted WMD of 2h-PG was -0.314 (95% CI: -1.599, 0.972; *P*=0.632) without notable heterogeneity (*I* ^2 =^ 38.9%). The adjusted WMD of FPG in the three RCTs was inconsistent and the result of the pooled adjusted WMD was imprecise. The evidence grading of changes in 2h-PG should also be considered as moderate (**Table 2**).

## 4 Discussion

The global epidemic of T2DM is an alarming cause of mortality and disability. Some physicians agree that herbal medicine and its formulations are safe and useful in treating persons with T2DM in many countries (48–50). The aim of our study was to evaluate the adjuvant hypoglycemic effect of jinlida granules in the treatment of T2DM and to explore the source of heterogeneity between these RCTs.

Nineteen of the 22 included RCTs were assessed as high risk of bias mainly because almost all the RCTs with high risk of bias did not mention the process of allocation concealment or blinding through the use of placebo. The results of most RCTs with high risk of bias showed a significant hypoglycemic effect of jinlida granules and the effects of lowering HbA1c and 2h-PG became more pronounced with the year of publication. The absence of blinding and insufficient randomization will result in between-trial heterogeneity and exaggeration of the treatment effect in RCTs (51–53). Considering the presence of methodological heterogeneity between RCTs with high risk of bias and RCTs with low risk of bias. We only pooled the data of the three RCTs with low risk of bias for outcomes.

Initially, the result of our meta-analysis did not show a significant hypoglycemic effect of jinlida granules. Notable statistical heterogeneity was present among the three RCTs (*I* ^2 =^ 68.2% for HbA1c; *I* ^2 =^ 77.4% for FPG; *I* ^2 =^ 75.7% for 2h-PG). Subsequently, efficacy after treatment in each RCT was adjusted according to the levels of baseline. Notable statistical heterogeneity was absent among the three RCTs (*I* ^2 =^ 0 for HbA1c; *I* ^2 =^ 29.1 for FPG; *I* ^2 =^ 38.9% for 2h-PG). It should be considered that difference at baseline is another source of methodological heterogeneity. The HbA1c-lowering effect of jinlida granules was statistically found. A significant effect of jinlida granules was still not statistically found in lowering FPG and 2h-PG. An insufficient number of RCTs and the small sample size resulted in imprecise results of our meta-analysis, which may be altered by future RCTs with low risk of bias. Additionally, statistical significance does not represent clinical significance in superiority trials (54, 55). Evidence grading of changes in HbA1c, FPG, and 2h-PG should be considered as moderate (GRADE system) and be interpreted cautiously.

Our study is the first systematic review and meta-analysis registered in PROSPRO to assess the adjuvant hypoglycemic effect of jinlida granules on metformin. The clinical heterogeneity of RCTs in our study was lower than that of the previous similar system review and meta-analysis due to the different inclusion criteria in the previous one (jinlida granules + hypoglycemic agents vs. hypoglycemic agents). In addition, the methodological heterogeneity between RCTs with low risk of bias and RCTs with high risk of bias is notable. We only pooled the data of the three RCTs with low risk of bias for outcomes instead of pooling them all (56). All RCTs in our study did not share a common-effect size, which means fixed-effect model is not appropriate for our meta-analysis (57). Effect values were analyzed using random-effects model instead of fixed-effect model. Many related RCTs have been published in the last three years. Our study updated these new RCTs to provide the latest information for rational decision by clinicians.

### 4.1 Limitations of the study

There are several limitations in our study. First, RCTs with low risk of bias were inadequate to accomplish sensitivity analysis and publication bias tests and to present robust results. Second, it is valuable to explore the heterogeneity among different subtypes of T2DM. RCTs with low risk of bias recruited participants with T2DM but not QYDT2DM. It was unable to achieve investigations of heterogeneity about different subtypes of T2DM. Third, we did not include gray literature data, which may contain useful data that could affect the outcome of the meta-analysis. Fourth, estimation of adjusted WMD reduces the test power of our meta-analysis. The quality evidence of the GRADE system for the outcomes in our meta-analysis was moderate and should be interpreted cautiously.

### 4.2 Implication of our study for TCM

#### 4.2.1 Suggestions for design of future RCTs about TCM

Many RCTs about TCM are of low quality (58). There are several suggestions for future RCTs evaluating the efficacy of TCM. Firstly, the future RCTs about this topic should be designed and described in detail to minimize the risk of bias in the following progress: how to generate the random sequence and to conceal the allocation of intervention groups or comparison groups; how to perform the blinding for participants and researchers who may influence the results of the trials; how to cope with the loss of participants in the trials; Whether the protocol of the RCTs has been registered in the China Clinical Trials Registry (http://www.chictr.org.cn). Secondly, many RCTs about TCM only state that there was no statistical difference in baseline characteristics between the intervention group and comparison group without detailed data. Differences at baseline are an important resource of methodological heterogeneity. It is necessary that data of baseline characteristics should be described in detail (mean with standard deviation or median with quartiles). Thirdly, whether the RCTs completed written informed consent and fulfilled ethical approval and the Declaration of Helsinki. Terms that stigmatize people with diabetes, such as diabetes patients or diabetic patients, should be replaced by persons with diabetes (59, 60).

#### 4.2.2 Suggestions for clinical practice and policy of TCM

T2DM is a heterogeneous group of disorders. Syndrome differentiation of TCM provides a new path for the classification of T2DM and is a prerequisites for the efficacy of TCM (61, 62). However, most RCTs have evaluated the adjuvant hypoglycemic effect of jinlida granules without syndrome differentiation of TCM. According to the package insert of jinlida granules and guidelines of T2DM, the indications of jinlida granules are not all subtypes of T2DM but rather QYDT2DM, which is only a subset of T2DM (62–64). It is obvious that jinlida granules are not suitable for persons with some other subtypes of T2DM such as yin-yang deficiency pattern and damp-heat trapping spleen pattern. Diagnosis and treatment guidelines of TCM should emphasize that physicians to prescribe Chinese patent medicines based on syndrome differentiation of TCM. RCTs that assess the effectiveness of TCM should be designed to take into account syndrome differentiation of TCM. In order to objectively evaluate hypoglycemic effect of statistically found jinlida granules, it is urgently needed that high-quality RCTs evaluating the hypoglycemic effect of jinlida granules in the treatment of QYDT2DM.

## 5 Conclusion

The adjuvant hypoglycemic effect of jinlida granules on adult Chinese persons with T2DM was statistically found in lowering HbA1c and was not statistically found in lowering FPG and 2h-PG. Evidence grading should be considered as moderate and the results should be interpreted cautiously. Whether the efficacy of HbA1c-lowering is related to clinical significance remains to be investigated in future RCTs. Differences in HbA1c, FPG and 2h-PG at baseline and high risk of bias were important source of heterogeneity between these RCTs. In order to objectively evaluate the efficacy of jinlida granules on T2DM, it is urgently needed that high-quality RCTs evaluating the hypoglycemic effect of jinlida granules in the treatment of QYDT2DM.

## Data availability statement

The original contributions presented in the study are included in the article/**Supplementary Material**. Further inquiries can be directed to the corresponding author.

## Author contributions

XZ designed the study, reviewed and revised the manuscript. XZ and LL independently completed the reports search, data extraction, quality assessment, and meta-analysis. All authors contributed to the article and approved the submitted version.

## Funding

The study was funded by Initial Scientific Research Fund for High-Level Talents of Chengde Medical University (201809 and 202204).

## Conflict of interest

The authors declare that the research was conducted in the absence of any commercial or financial relationships that could be construed as a potential conflict of interest.

## Publisher’s note

All claims expressed in this article are solely those of the authors and do not necessarily represent those of their affiliated organizations, or those of the publisher, the editors and the reviewers. Any product that may be evaluated in this article, or claim that may be made by its manufacturer, is not guaranteed or endorsed by the publisher.

## Supplementary material

Supplementary Figure 1 Weighted Mean Difference of HbA1c in each RCTs with High Risk of Bias (Jinlida vs. Control).

Supplementary Figure 2 Weighted Mean Difference of 2h-PG in each RCTs with High Risk of Bias (Jinlida vs. Control).

Supplementary Figure 3 Weighted Mean Difference of FPG in each RCTs with High Risk of Bias (Jinlida vs. Control).
