# Supplementary figures and images for: Treatment of type 2 diabetes mellitus using the traditional Chinese medicine Jinlida as an add-on medication: A systematic review and meta-analysis of randomized controlled trials
Source: Front Endocrinol (Lausanne). 2022 Oct 17;13:1018450. doi: 10.3389/fendo.2022.1018450 (PMC9618612; doi:10.3389/fendo.2022.1018450)

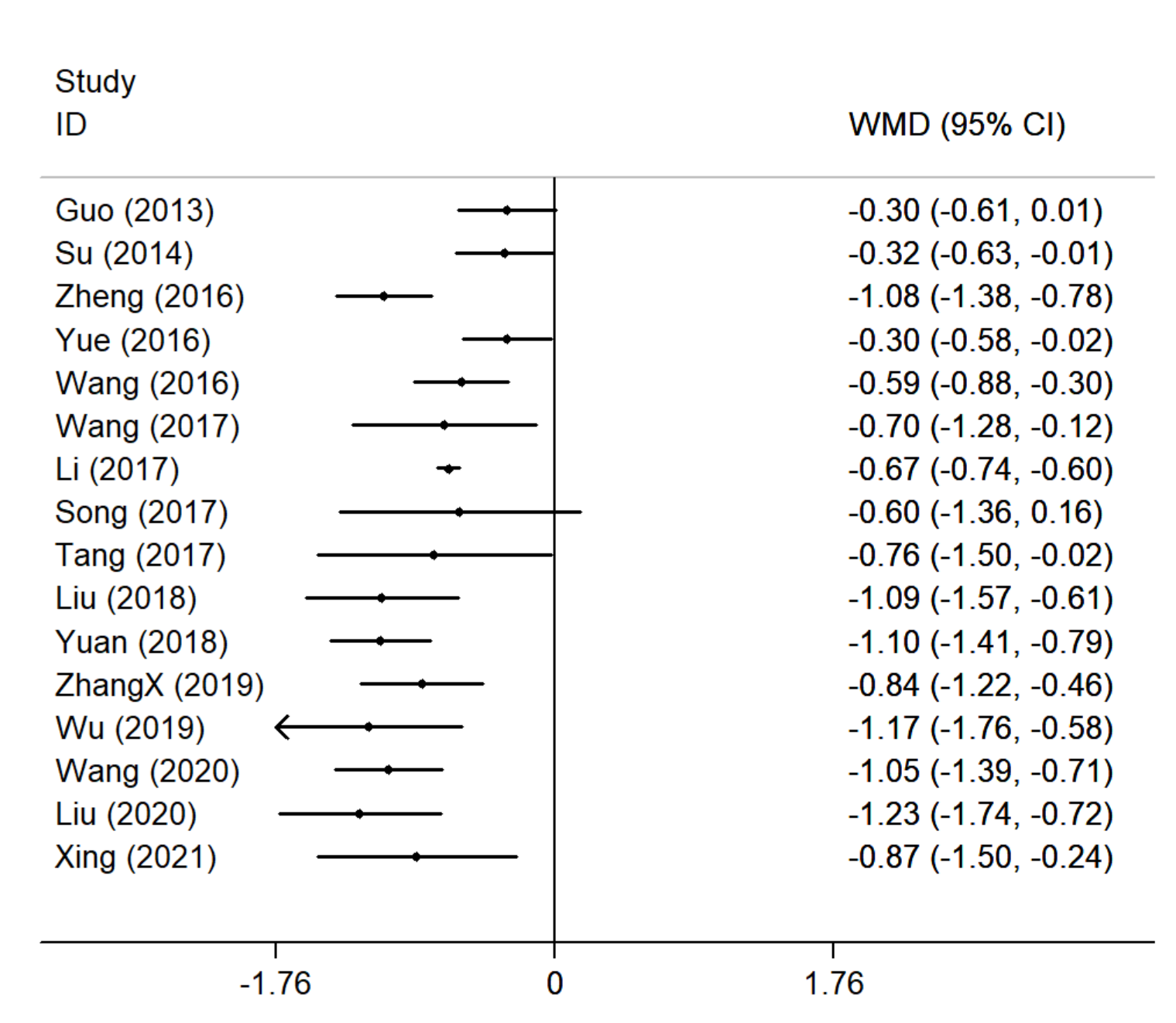

Supplement: Supplementary Figure 1 — Weighted Mean Difference of HbA1c in each RCTs with High Risk of Bias (Jinlida vs. Control). [file Image_1.tif]

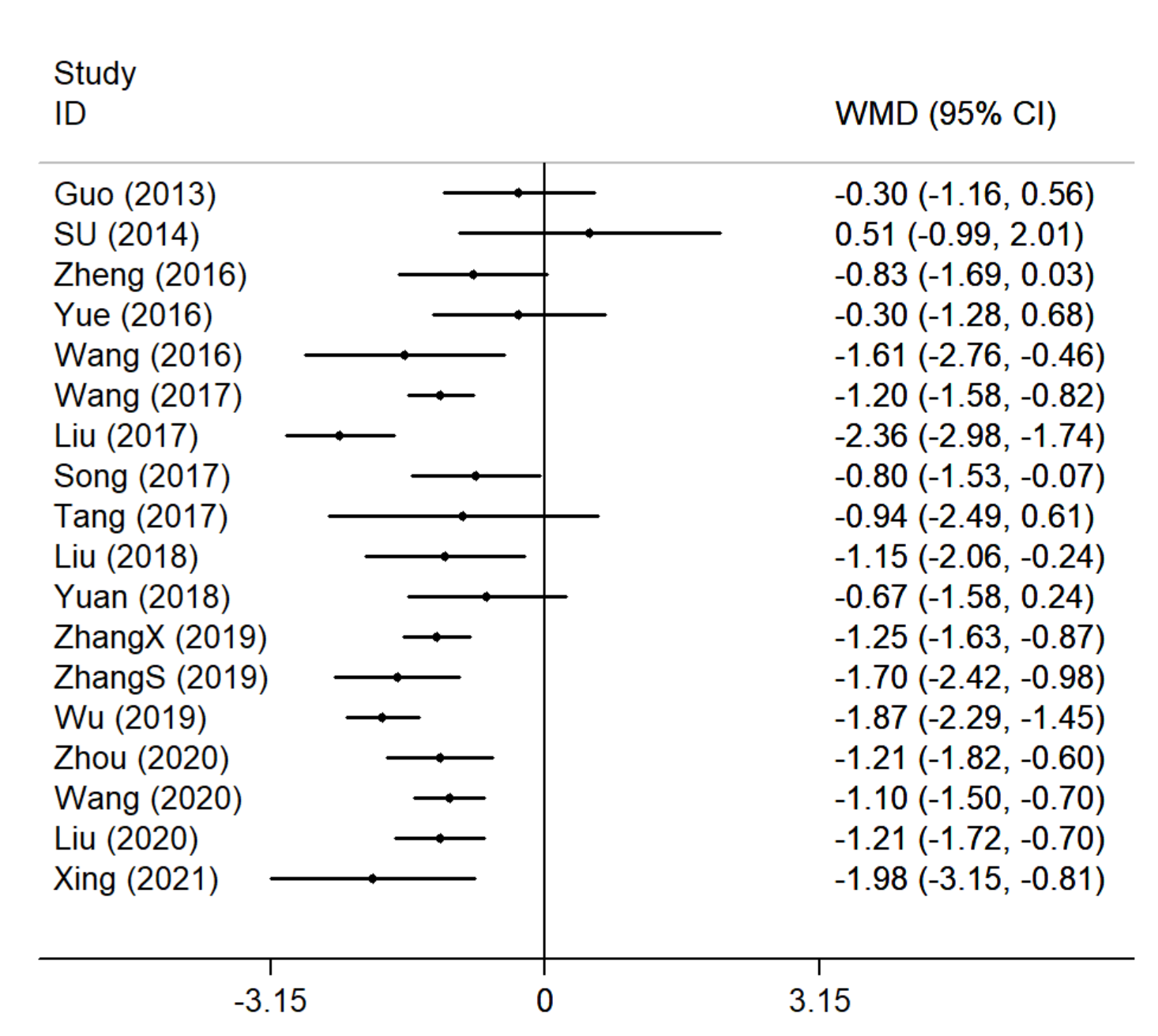

Supplement: Supplementary Figure 2 — Weighted Mean Difference of 2h-PG in each RCTs with High Risk of Bias (Jinlida vs. Control). [file Image_2.tif]

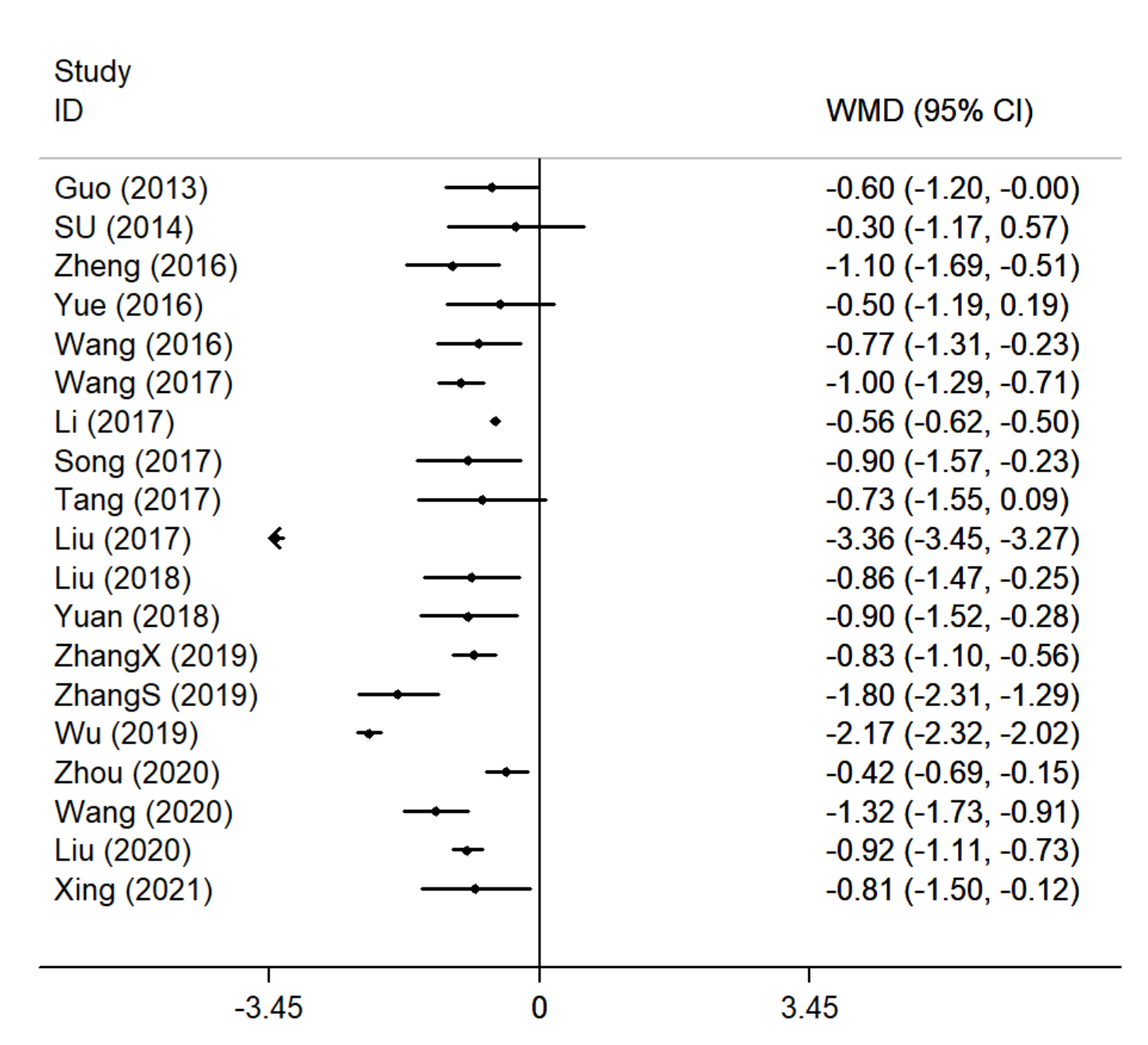

Supplement: Supplementary Figure 3 — Weighted Mean Difference of FPG in each RCTs with High Risk of Bias (Jinlida vs. Control). [file Image_3.tif]
